# Mammographic density: a potential monitoring biomarker for adjuvant and preventative breast cancer endocrine therapies

**DOI:** 10.18632/oncotarget.13484

**Published:** 2016-11-21

**Authors:** Michael S. Shawky, Hilary Martin, Honor J. Hugo, Thomas Lloyd, Kara L. Britt, Andrew Redfern, Erik W. Thompson

**Affiliations:** ^1^ Department of Head and Neck and Endocrine Surgery, Faculty of Medicine, University of Alexandria, Egypt; ^2^ Department of Surgery, University College Hospital, London, UK; ^3^ School of Medicine and Pharmacology, University of Western Australia, and Department of Medical Oncology, Fiona Stanley Hospital, Perth, Western Australia, Australia; ^4^ Institute of Health and Biomedical Innovation and School of Biomedical Sciences, Queensland University of Technology, Australia; ^5^ Translational Research Institute, Brisbane, Australia; ^6^ Department of Radiology, Princess Alexandra Hospital, Brisbane, Australia; ^7^ The Sir Peter MacCallum Department of Oncology, University of Melbourne, Melbourne, Australia; ^8^ Peter MacCallum Cancer Centre, Melbourne, Australia; ^9^ Department of Anatomy and Developmental Biology, Monash University, Melbourne, Australia; ^10^ Department of Surgery, University of Melbourne, St Vincent’s Hospital, Melbourne, Australia

**Keywords:** mammographic density, breast cancer, endocrine therapy, predictive biomarker, surrogate

## Abstract

Increased mammographic density (MD) has been shown beyond doubt to be a marker for increased breast cancer risk, though the underpinning pathobiology is yet to be fully elucidated. Estrogenic activity exerts a strong influence over MD, which consequently has been observed to change predictably in response to tamoxifen anti-estrogen therapy, although results for other selective estrogen receptor modulators and aromatase inhibitors are less consistent. In both primary and secondary prevention settings, tamoxifen-associated MD changes correlate with successful modulation of risk or outcome, particularly among pre-menopausal women; an observation that supports the potential use of MD change as a surrogate marker where short-term MD changes reflect longer-term anti-estrogen efficacy. Here we summarize endocrine therapy-induced MD changes and attendant outcomes and discuss both the need for outcome surrogates in such therapy, as well as make a case for MD as such a monitoring marker. We then discuss the process and steps required to validate and introduce MD into practice as a predictor or surrogate for endocrine therapy efficacy in preventive and adjuvant breast cancer treatment settings.

## INTRODUCTION

Breast cancer (BC) is the most common cancer in women and the second leading cause of cancer-related death among females in the US according to 2016 statistics [1]. Latest global cancer statistics show that 1.7 million BC diagnoses and 522,000 BC-related deaths were recorded in 2012, with a 20% increase in the BC incidence rate and 14% increase in the BC-related mortality rate, in comparison with 2008 estimates [2]. Such striking statistics highlight the importance of optimizing the prevention and treatment of this disease.

Approximately 60-75% of BCs are hormone receptor positive, expressing estrogen receptor (ER), progesterone receptor (PR) or both [3]. In keeping with this, cumulative estrogen exposure has consistently been demonstrated to elevate BC risk such that doubling of serum estrogen raises BC relative risk by 1.29 fold [4]. Factors that modulate estrogen exposure throughout life, including endogenous elements such as age of menarche and menopause, breast feeding and post-menopausal obesity, and exogenous elements such as oral contraceptives and hormone replacement therapy (HRT), all influence BC risk in a direction predictable by their impact on estrogen levels [5].

Predictably then, endocrine therapy (ET) may be used both to reduce the risk of contracting such cancers [6, 7] as well as to attenuate the risk of their return after surgery for early stage disease [8]. A choice of agents needs to be made, particularly between the selective ER modulator (SERM) tamoxifen and the aromatase inhibitors (AIs), which suppress post-menopausal estrogen synthesis. This is in the context of treatment over 5 to 10 years, sometimes with significant toxicity and with no method of ascertaining whether a particular treatment strategy is effective or futile until the advent of BC occurrence/recurrence or otherwise. For this reason, numerous BC biomarkers have been studied to guide both the utility of ET overall as well as agent choice, although to date only the presence of ER and PR are used to predict overall benefit in the clinic and no biomarker guides agent choice.

On this background, mammographic density (MD) shows significant promise as a tool to refine ET decisions. MD is an imaging parameter that reflects the relative amounts of various tissue elements in the breast. It is well established that radiographic MD histologically corresponds to higher fractions of stroma and epithelium relative to adipose tissue [9]. It has also been demonstrated to correlate directly and incrementally with BC risk [10] in such a way that for every 3-6% MD rise, relative BC risk increases by 10% [11]. In addition to demonstrating utility in predicting the initial risk of BC [12], MD at diagnosis also correlates with subsequent risk of local but not distant relapse [13].

Higher MD, which correlates with increasing BC risk, has been documented in post-menopausal women receiving exogenous estrogen as a replacement therapy [14, 15], as well as among non-HRT users with higher levels of endogenous estrogen [16]. In addition, more frequent expression of ERα has been found in the stroma of the risk-associated higher MD mammary tissue [17]. Thus, an intrinsic causal link appears likely within this triad of higher estrogen activity, high MD and elevated BC risk. It therefore follows that gaining a greater understanding of the underlying pathobiology linking MD and BC, and how estrogens and anti-estrogens may broker that link, could give us new insights into improved therapeutic approaches against the disease beyond prediction of risk.

As defined by the Biomarker Definition Working Group of the National Institute of Health, USA, a biomarker is any objectively measurable characteristic that could indicate an underlying physiological or pathological process (e.g. BC risk) or evaluate the response to an intervention (e.g. ET) [18]. Two concepts should be distinguished, that of a prognostic marker, which informs about the native risk of certain outcomes in the absence of any intervention, to be differentiated from a predictive marker which predicts response to an intervention [19]. Importantly, the FDA considers surrogate markers in the approval process, measurable parameters that may be used as a substitute for clinically relevant endpoints in interventional trials, essentially synonymous with predictive markers [20]. For a biomarker to be validated as “predictive”, correlation with outcome needs to be evaluated in clinical trials treating the disease where the intervention is randomly allocated to the subjects, ideally with the biomarker study forming part of the prospective design [21]. A control group is essential for full predictive biomarker evaluation, excluding the possibility that correlations between biomarker and outcome are not purely due to a prognostic effect.

There is a surfeit of commonly employed prognostic biomarkers used to gauge the risk of both BC incidence, such as family history and prior breast biopsy [22]; or relapse, such as lymph node involvement, size and grade of tumour [23]. In contrast, there is a dearth of predictive markers for ET efficacy in these scenarios. In addition to data indicating predictive ability, a potential biomarker that could indicate ET efficacy would ideally fulfil the following criteria: 1) *biologically plausible* [24], that is an intrinsic link to BC therapeutic biology would account for the prediction of ET-mediated protective effect, 2) *serially measurable*, such that it can be practically used to indicate whether the ET-mediated risk-modifying process is proceeding in a favourable way relatively early in therapy, when tailoring of the preventive intervention can still be undertaken i.e. before BC occurrence or recurrence, and 3) *applicable* to both the preventive and adjuvant settings.

Immunohistochemical (IHC) biomarkers such as ER expression [25] and STAT5 expression [26] are indeed helpful for deciding which patients may benefit from ET on pathogenesis related grounds and thus fulfil criterion 1, although they do not provide guidance on ET agent choice. However, being inherent characteristics of the initial tumour, such biomarkers are not useful as monitoring tools as change cannot be measured to reflect ET action and they are not useful in the preventive setting as there is no actual tumour to assess. Although MD has plausible biological links to breast cancer risk (criterion 1), unlike these biomarkers it cannot be used to specifically select patients likely to benefit from ET prior to treatment in the adjuvant setting. In contrast, however, MD may be measured through the treatment period, with MD change potentially reflecting ET efficacy (criterion 2). Additionally, being a character of the “breast” rather than the “tumour” with pathogenic links to BC risk, MD change may be used to predict ET efficacy in the preventive setting (criterion 3) and serial monitoring of the unaffected breast maintains MD utility in the adjuvant setting after surgical excision of the cancerous breast tissue (criterion 3). Thus, MD is intuitively a compelling biomarker for monitoring the response to ET in patients selected for therapy. Such therapy may be dictated by MD stratification of risk in primary prevention and according to ER and PR status in the adjuvant setting.

In this article, we have reviewed the MD changes in relation to the spectrum of ETs employed in different BC scenarios to explore whether evidence to date supports this hypothesis and, if this proves to be the case, to ascertain what further research is required to bring this into routine practice. Specific aims of the review are to:

1. Look at degree and timeframe of MD change with currently employed ETs.
2. Assess the utility of serial MD measurements for predicting the primary preventative benefit of chemoprophylaxis in females with elevated BC risk.
3. Assess the effectiveness of serial MD measurement in the follow-up of females with surgically excised early breast cancer on adjuvant ET to prevent recurrence.
4. Explore the mechanisms connecting MD with breast cancer risk and prevention to identify new biological avenues of protection from adverse breast cancer outcomes.

## RESULTS

### MD change on currently employed ET

A total of 19 publications recorded MD changes across periods of ET. Table 1 summarizes MD changes on SERMs. With regards to the seven studies exploring changes on tamoxifen, all of which were in populations including pre-menopausal women [27–31], by far the largest study was the IBIS I prevention study accounting for more than two thirds of patients. Significant reductions in all sub-groups were seen on tamoxifen relative to placebo; 7.9% v 3.5% at 18 months (*p* < 0.001) and 13.7% v 7.3% at 54 months (*p* < 0.001) [27]. The three other trials with placebo or control arms [28–30] all showed significant MD reductions on tamoxifen despite low participant numbers. Two other recent retrospective studies have also demonstrated an annual MD reduction in tamoxifen-treated women [32, 33]. Of interest, the small single study looking at subsequent MD change after tamoxifen completion showed small increases in MD post-tamoxifen in 48% of cases, albeit of unknown prognostic significance [31].

**Table 1:** Studies investigating MD changes in response to use of SERMs

Study		Subjects	Protocol					Results (PMD Change)			
			Mammography		Agent and dose	Time	N	PMD (%)		*p* value	
Reference	Trial Type	Age (yr)				On		Baseline	Finish	versus baseline	versus control
		Clinical Scenario	Measure	Post-Start		Therapy					
**Tamoxifen**											
Brisson 2000[28]	RCT	≥35 Prevention	PMD, Wolfe	41 m	Placebo Tamoxifen 20mg	60 m	33 36	60.5 60.3	51.1 56.7	NR NR	NA 0.01
Chow 2000[29]	RCT	36-74 Prevention	PMD Boyd, BIRADS	22 m	Control Tamoxifen 20mg	24 m	20 27	29.7 31.9	29.6 29.2	0.88 0.0007	NA NR
Konez 2001[31]	Retro cohort	31-81 Adjuvant post-BC	MD Category	60 m	Tamoxifen 20mg	60 m	24	20.8%*		0.06	NA
Cuzick 2004[27]	RCT	35-70 Prevention	PMD, Boyd	54 m	Placebo Tamoxifen 20mg	60 m	430 388	42.6 41.9	35.3 28.2	<0.001 <0.001	NA <0.001
Meggiorini 2008[30]	Retro cohort	41-78 Adjuvant post-BC	BIRADS	12 m	Control Tamoxifen 20mg	60 m	80 68	30%* 50% *		NR NR	NA 0.021
Howell 2015[33]	Retro cohort	33-46 Prevention	PMD	12 m	Tamoxifen	12m	105	49%^†^		NR	NA
Engman 2016[32]	Retro cohort	45-60 Adjuvant post-BC	VPD, DV	annually	Tamoxifen	36 m	379	11.6 64.7	17%** 0,90**	NR NR	NR NR
**Raloxifene**											
Freedman 2001[39]	RCT	45-60 Prevention Post-menopause	PMD	24m	Placebo Raloxifene 60mg Raloxifene 150mg	24 m	45 45 42	9.8 9.3 8.1	8.5 7.7 6.4	<0.02 <0.02 <0.02	NA NS NS
Christo- doulakos 2002[38]	RCT	41-67 Risk of osteoporosis	Wolfe	12m	Placebo Raloxifene 60 mg Tibilone 2.5mg	12 m	27 48 56	25.9 ^†^ 18.8^†^ 10.7^†^	0 ^††^ 6.3 ^††^ 10.7 ^††^	NR NR NR	NA 0.47 0.07
Cirpan 2006[37]	Retro cohort	43-58 Osteoporosis	BIRADS	12m	Raloxifene 60mg	12 m	55	MD category increase in one patient. Otherwise, no change		0.32	NA
Eng-Wong 2008[40]	CT	35-47 Prevention	PMD	24 m	Raloxifene 60mg	24 m	27	38	41.5	NS	NA
Eilertsen 2008[36]	RCT	45-65 Prevention	PMD (volume)	12 week	Raloxifene 60 mg Low dose HRT Standard dose HRT	12 week	44 44 45	7.7 8.6 8.3	8.1 11.2 10.6	0.09 <0.0001 <0.0001	NA NA NA
Nielsen 2009[35]	RCT	55-80 Risk of osteoporosis	PMD BIRADS	24m	Tibolone Raloxifene 60 mg Estradiol 0.014 wk,	24 m	45 135 135	7.5 16 16	8.1 18 20	0.9 NS <0.05	NA NA NA
Harvey 2013[34]	RCT	40-75 Risk of osteoporosis	PMD	24m	Placebo BZA 20mg/E 0.45mg BZA 20mg/E0.625 mg Raloxifene 60 mg	24 m	126 129 105 125	26.1 26.5 25.3 27.2	25.7 26.1 25.2 27	significant significant NS NS	NA NS NS NS

* % pts with MD category reduction, ^†^ % pts with MD decrease, **annualized MD reduction, ^††^ % pts with MD increase

RCT: Randomised Controlled Trial, Retro cohort: Retrospective cohort, BC: Breast Cancer, PMD: Percent Mammographic Density, BIRADS: Breast Imaging-Reporting and Data System, VPD: Volumetric Percent Density, DV: Dense Volume, m: month, HRT: Hormone Replacement Therapy, BZA: Bazedoxifene, NS: Not Significant, NR: Not Reported, NA: Not Applicable

For the alternative SERM raloxifene, six of seven studies have been in post-menopausal patients [34–39]. Although numbers have been low, and comparisons sometimes made with patients on HRT rather than placebo [36], none have shown significant MD changes relative to placebo or baseline in single arm trials. Further, two studies have shown trends to increases rather than decreases of MD on the drug [35, 38]. The single pre-menopausal study showed no impact of raloxifene on MD [40]. In keeping with this the head-to head STAR trial, comparing tamoxifen to raloxifene for primary prevention showed a significant modestly inferior protection with raloxifene compared to tamoxifen, although whether individuals experiencing an MD increase responded poorly is unknown [41].

Despite the proven therapeutic efficacy of aromatase inhibitors (AIs) in the adjuvant setting [42], MD has not been observed to change consistently or significantly in response to these agents (Table 2). Of six relevant studies, three uncontrolled studies showed MD reductions with two reaching significance [32, 43, 44]. Three further trials with control arms demonstrated no MD reduction relative to untreated patients, although numbers in all trials were low [45–47]. To summarize, contrary to the case with tamoxifen, a convincing case for using MD to monitor the effects of AIs and other SERMs is not evident from this assembled data.

**Table 2:** Studies investigating MD changes in response to use of AIs

Study		Subjects	Protocol					Results (PMD Change)			
			Mammography		Agent and dose	Time	N	PMD (%)		*p* value	
Reference	Trial Type	Age (year)				On		Baseline	Finish	versus baseline	versus control
		Clinical Scenario	Measure	Post-Start		Therapy					
Vachon 2007[45]	RCT	30-84, post-tamoxifen Adjuvant post-BC	PMD	12 m	Placebo Letrozole 2.5 mg	60 m	33 35	20 18.5	19 16.9	NR NR	NA 0.58
Cigler 2010[46]	RCT	Post-menopausal Prevention	PMD Boyd, BIRADS	24 m	Placebo Letrozole 2.5mg	12 m	16 27	40 39.6	38.7 39.6	0.71 0.99	NA 0.69
Cigler 2011[47]	RCT	Post-menopause Prevention	PMD Boyd, BIRADS	12 m	Control Exemestane 25mg	12 m	31 34	36.5 33.9	37.1 34.5	NR NR	NA 0.91
Smith 2012[43]	CT	Mean 58.9 Prevention	PMD	12 m	Letrozole 2.5mg	12 m	16	27.7	23	0.036	NA
Gatti-Mays 2016[44]	Phase II trial	Post-menopausal Prevention	PMD	24 m	Exemestane 25mg	24 m	35	32.5	28.4	0.009	NA
Engman 2016[32]	Retro cohort	58-71 Adjuvant post-BC	VPD DV	annually	AIs	36 m	425	7.2 51.9	0.19%* 0.52*	NR NR	NR NR

^*^annualized MD reduction

RCT: Randomised Controlled Trial, Retro cohort: Retrospective cohort, BC: Breast Cancer, PMD: Percent Mammographic Density, BIRADS: Breast Imaging-Reporting and Data System, VPD: Volumetric Percent Density, DV: Dense Volume m: month, NR: Not Reported, NA: Not Applicable

### Correlations between MD change and primary prevention efficacy

From our review of the data, only one study has directly linked tamoxifen-induced MD reduction to the subsequent risk of developing BC; the report of Cuzick and colleagues on a nested study from the IBIS I trial of tamoxifen *versus* placebo in women at high risk of the disease [48]. Here they report a significant 63% BC risk reduction among tamoxifen users having greater than 10% MD reduction (MDR), compared to no risk reduction if MDR was less than 10% (odd ratios: 0.37 *vs*. 1.13), as assessed visually in 12-18 month post-treatment mammograms [48, 49]. No literature was identified regarding MD change in the IBIS II [7] or MAP.3 [42] studies, which have employed anastrozole and exemestane respectively *versus* placebo for primary BC prevention. Howell et al subsequently reproduced this spectrum of MD changes on preventative tamoxifen, though highlighted the difficulty of assessing MD consistently in general radiological practice and suggested introducing volumetric methods for clinical usage [33].

### Correlations between MD change and adjuvant ET benefit

Before reviewing the predictive impact of MD change on adjuvant ET efficacy, it is worthwhile to consider first the prognostic impact of MD baseline present at the time of BC diagnosis. A lower MD has been reported in most studies to be linked to better BC outcomes in terms of lower risk of local recurrence [13, 50], death from all causes [51] and risk of second BC [52], although none have shown a significant association to the risk of distant metastasis or BC-specific mortality. Baseline MD is thus prognostic for certain parameters of outcome.

Turning to the prediction of ET benefit in the adjuvant setting, seven studies have directly assessed BC outcome measures in relation to ET-induced MD changes (Table 3). Three case-control studies [53–55] and three retrospective cohorts [56–58] have demonstrated an association between MD reduction in response to ETs and better BC outcomes, in terms of BC-related death, BC recurrence and contra-lateral primary. Risk reductions were uniformly significant and robust for disease free survival (DFS) (0.36-0.52) and overall or BC specific survival (0.44-0.50), although these trials involved mostly tamoxifen and were mostly in younger women. One study failed to demonstrate an association between MD reduction and lowering of recurrence events in response to exemestane or tamoxifen in post-menopausal women, although the MD changes were small [59]. Contrary to this, the studies of Kim et al [57] and interim analysis of a study by members of our group [58], found MD reduction to be a predictor of outcome in mixed tamoxifen and AIs treated cohorts.

**Table 3:** Studies investigating BC risk/outcome modification in relation to ET-induced MD changes

Study		Subjects	Protocol						Results		
Reference	Type	Age (yrs) Menopausal status	Mammography			Event	Medication	N	BC-Related Outcomes		
			Method	Time on Rx	MD Change Categories				HR (MDR v not)	95% CI	*p*
**Preventive - Women at high BC risk**											
Cuzick 2011[48]	Case cont within RCT	35-70 Mixed	PMD (PDA)	12-18m	MDR ≥10% v no decrease	BC risk	Tamoxifen	507	0.32	0.14-0.72	0.001
**Adjuvant – Hormone receptor positive early breast cancer**											
Kim 2012[57]	Retro cohort	25-78 Mixed	PMD	13 m	MDR ≥ 10% v MDR < 0%	BCR	Tamoxifen or AI	1065 (938 invasive)	0.44	0.22-0.91	0.027
Ko 2013[56]	Retro cohort	25-78 Mixed	BIRADS	19 m	MDR by 1+ BIRAD category v no change BIRAD category	BCR	Tamoxifen	1066 (932 invasive)	0.36	0.18-0.70	0.003
Li 2013[54]	Case cont	Median 62-63 Post-menopause	ADA	17 m	MDR > 20% v MDR < 10%	BCSM	Tamoxifen	474	0.5	0.27-0.93	0.017
Sandberg 2013[55]	Case cont	NR Post-menopause	PMD	19 m	MDR ≥ 10% v MDR < 10%	CBC risk	Tamoxifen	87	0.52	0.18-1.51	NS
Nynate 2015[53]	Case cont	32-87 Mixed	PMD (PDA)	12m	Tertiles highest MDR (>8.7 %) v lowest MDR (<0.5 %)	BCSM	Tamoxifen	349	0.44	0.22-0.88	0.005
Martin 2015[58]	Retro cohort	25-96 Mixed	PMD	11-24 m	MDR > 20% v MDR < 0%	BCR	Tamoxifen or AI	921	0.45	0.25-0.80	0.006
Van Nes 2015[59]	Retro cohort within RCT	45-91 Post-menopause	PMD (PDA)	24 m	Pts divided into 6 categories No density change on Rx	BCR	Tamoxifen or Exemestane	377	NR No density change	NR	NR

RCT: Randomised Controlled trial, Retro cohort: Retrospective cohort, MDR: Mammographic Density Reduction, PMD: Percent Mammographic Density, BIRADS: Breast Imaging-Reporting and Data System,PDA: Percent Dense Area, ADA: Absolute Dense Area, BCSM: Breast Cancer Specific Mortality, BCR: Breast Cancer Relapse, CBC: Contralateral Breast Cancer, AI: Aromatase Inhibitor, HR: Hazard ratio NR: Not Reported

Findings from these studies suggest that MD change could be a valuable biomarker for predicting the impact of adjuvant tamoxifen on ER positive BC outcomes in younger women, with more heterogeneous evidence in older post-menopausal women, particularly those on AIs.

### Biological processes underlying MD change, BC risk and ET action

Recent genetic studies have identified certain single nucleotide polymorphisms (SNPs) with overlapping effects on both BC risk and MD, suggesting that the BC risk conferred by these loci is at least partly mediated through their effects on MD [60]. Polymorphisms of genes involved in epidermal growth factor (EGF), ER and insulin-like growth factor 1 (IGF1) signalling, cell proliferation, and migration are included [61]. Furthermore, MD changes in perimenopausal women of different racial descents have been linked to SNPs in genomic loci encoding enzymes controlling sex steroid metabolism and ERs. Since the former act upon and the latter are expressed in breast tissue, such SNPs could therefore impact BC risk through MD modulation *via* the estrogen/ER interaction [62]. Additionally, recent analysis of known BC susceptibility genes has identified two novel MD loci in 6q25.1 region, one of which is in the TAB2 gene [63], a potential driver of tamoxifen resistance [64]. Reduced tamoxifen effectiveness, mediated through these loci, may explain the higher risk of local relapse and contralateral primary BC associated with higher MD.

Turning to potential proteomic drivers of risk, high MD tissue shows higher expression of proteins involved in angiogenesis, inflammation, proliferation and estrogen synthesis, whereas low MD tissue shows higher expression of effectors of cell cycle arrest and estrogen inactivation [65]. Fibrous stroma of women at high risk of BC was noted to very frequently express ERα regardless of MD status, whereas frequent expression of PRα was found in the stroma of high MD compared to low MD mammary tissue, and is thus also pathogenically implicated [17].

Considering mammographically dense tissue from the perspective of whole tissue cellular interactions, the influence of MD on breast cells at risk, roles of stromal cells that appear to confer risk, and adipose cells that may confer protection are key questions [60].

Tamoxifen is the most extensively studied ET in both preventive and adjuvant settings for BC and also the drug with the most consistent links between induced MD change and BC. Therefore, it is worthwhile reviewing the established and possible mechanisms through which tamoxifen could impact both MD and BC. In terms of tamoxifen effects on breast tissue, the drug was noted to cause a reduction in the stromal component of biochamber-implanted HMD tissue, which was associated with reduction of MD [66]. In addition to contributing to an HMD status [67], a high stromal density contributes to a higher BC risk in animals [68, 69]. Thus, tamoxifen-induced reduction of stromal density could reduce BC risk concomitantly with its demonstrated MD lowering effect. Similarly, a reduction in the absolute epithelial mass has been observed in response to tamoxifen treatment in rodents. Such reduction was concomitant with MD reduction [70] and possibly with lower BC events. Additionally, the proliferative state of epithelial cells may affect both MD and the probability of cancer-initiating genetic damage [71]. However, the role of tamoxifen-mediated reduction of stromal and epithelial components in causing reduction of MD-associated breast carcinogenesis is speculative and needs further confirmatory studies.

Looking at specific signalling pathways, a number of pathways that mediate the pathogenesis of BC and MD have been identified as targets for tamoxifen action. Tamoxifen has been reported to enhance expression of transmembrane receptor CD36 [72], which was found to be repressed both in HMD tissue and in BC [73]. Suppression of Hedgehog signalling in response to tamoxifen has been demonstrated to remodel the stromal micro-environment [74] through down-regulation of stromal fibroblast proliferative activity [75]. A higher proliferative rate of stromal fibroblasts has been implicated in HMD status [76] and higher rate of cancer initiation as well [77]. Tamoxifen was also reported to interfere with IGF1 receptor (IGF-1R) signalling [78]; the expression of which has been found to increase in relation to both BC and increased MD [79, 80]. Similarly, tamoxifen has been known to reduce circulating IGF-1 [81], which was found to positively correlate with both BC risk [82] and HMD [83]. Furthermore, tamoxifen treatment of biochamber-implanted HMD tissue has been shown to alter Cox-2 expression in stromal cells [84], which has been found in other studies to be implicated in both MD and tumour initiation [85], adding another possible mechanism of tamoxifen reduction of MD and associated BC events.

## DISCUSSION

### MD modification on ET

Across the spectrum of available studies tamoxifen has been shown to reduce MD in a significant proportion of participants, which has allowed further analysis of these changes in relation to efficacy. The degree and direction of change on raloxifene is more heterogeneous with significant MD reduction not being seen in any study. Whether this represents a subtly different mechanism of action or lower potency and efficacy, as suggested by the previously referenced STAR trial, is unclear. Significant MD changes on AIs have not been frequently observed; which could suggest that AIs may exert their therapeutic effect through non-MD mediated mechanisms. Additionally, as AIs are only indicated as single agents in the postmenopausal setting where baseline MD tends to be low, this may also explain why their effects on MD are not significant. In keeping with this, most studies have observed that MDR was more substantial in women having initially higher MD [27, 86, 87]. Whether a longer period of observation is useful to capture more significant change is unknown, although a significant prolongation of time to acquisition of a usable efficacy prediction would obviously diminish clinical utility.

### MD change in premenopausal women

Both the likelihood and the magnitude of ET-induced MD reduction have been universally observed to be greater among pre-menopausal women, at least in part due to their higher baseline MD at study entry time, which is attributable to higher ovarian estrogen production. Additionally, pre-menopausal women are generally younger and tend to be more physically active, thus possibly having lower BMI. Both factors, younger age [56] and lower BMI [27], have been demonstrated in multivariate analysis to be significant independent predictors of greater MD reduction. It is thus expected that the potential for MD change as an ET-monitoring tool would be greatest among this subgroup of women.

### MD as a predictive surrogate in primary prevention

The uptake of tamoxifen in primary prevention by women at high risk of BC is low with less than 5% of high risk women utilising this approach [88]. Confirmation of the provocative findings relating to MD change and benefit seen in IBIS I in a second study utilising tamoxifen would assist in bringing MD assessment into clinical practice and could significantly improve preventative tamoxifen uptake. The optimal trial design would explore a strategy of continuation or discontinuation of preventative treatment based on MD change relative to continued therapy regardless of MD alterations, and would measure subsequent BC risk. Extension of research into prevention studies employing AIs such as IBIS II [7] or MAP.3 [42] would both clarify the role of MD change with this class of agent and add a valuable prediction tool across the future BC prevention landscape. If positive, this could particularly guide personalised prevention allowing tailoring towards the most effective agent for the individual; SERM or AI.

### MD modulation in adjuvant studies

Considering the observations that initial MD can predict the risk of second primary and local relapse, this supports the hypothesis that tamoxifen-induced MD reduction may, at least in part, reflect improvements in BC outcome through modulation of the effects of the local breast environment on developing or residual malignant cells.

Across the adjuvant tamoxifen studies, particularly in younger women, the predictive power of MD change appears consistent and robust. However, all studies to date have failed to account for important potential confounders. An important example of such confounders is chemotherapy treatment, which frequently induces menopause in treated pre-menopausal patients. The resultant reduction in ovarian hormonal output can significantly improve BC outcomes, as most recently evidenced by the SOFT trial in women still pre-menopausal after chemotherapy [89], and would also be expected to bring about a reduction in MD. In this regard it is noteworthy that the two largest studies by Kim et al [57] and Ko et al [56] had mean ages of 49 and 45 years and chemotherapy delivery rates of 77% and 68% respectively, suggesting that chemotherapy-induced menopause would have been a frequent occurrence. Chemotherapy-induced menopause should, therefore, be accounted for in correlative analyses of MD reduction and protective benefit to exclude this confounder as the driver of MDR-protective benefit correlation. Furthermore, older women ceasing hormone replacement therapy (HRT) at diagnosis could also experience prognostically important MD falls independent of ET effect. Interim analysis was recently presented of research undertaken by members of our group examining the predictive power of MD fall in the first 921 patients of a 1942 adjuvant ET-treated retrospective cohort [58]. Full data on chemotherapy-induced menopause and HRT cessation are available for this study cohort and will be factored into the final analysis.

The value of MDR as a predictor of AI efficacy is less clear. In the larger retrospective study of Kim et al [57], MDR had similar predictive power for AI and tamoxifen usage. The study by members of our group mirrored this; patients on tamoxifen and AIs experienced numerically close MDRs, which have been subsequently found to correlate significantly with DFS in the entire cohort, suggesting therefore that MDR could be predictive of AI efficacy as well. The detailed results however are yet to be reported in the final analysis [58]. In contrast, in the study of Van Nes et al of patients from the prospective TEAM study, infrequent MDRs and a lack of correlation with outcome were observed [59]. The reason for this heterogeneity is unclear. The most common AIs in general use, and used in the study of Kim et al, are the non-steroidal AIs (NSAIs) letrozole and anastrozole. In contrast, the negative study [59] employed exclusively exemestane. Inter-agent differences in effect on MD are thus a possibility although efficacy differences have not been seen in head-to-head trials between AIs. Exploring alternative explanations, it is noteworthy that 77% of patients in the study of Kim et al received chemotherapy and thus a proportion of patients may have received an AI following on from chemotherapy-induced menopause, a time at which MD often falls. Again in contrast, the TEAM study required patients to have confirmed menopause at study entry and hence may have been further from menopause with consequently lower MD, making resultant changes smaller on treatment. Additionally, high BMI can confound AI efficacy and also interact with MD. On the TEAM study, 61% of this older European-derived patient population was overweight or obese. BMI was not reported in the study of Kim et al, but might be expected to be significantly lower in a younger Korean population, raising the possibility that BMI might differentially modulate AI efficacy and explain inter-trial MD change discrepancies. Contrary to this, the BMI demographic in the Australian study by members of our group was more reflective of the TEAM population with 65% overweight or obese but still showing measurable MD falls in the entire cohort, including those on AIs.

It is also worth considering the biology behind the association between MDR and BC outcome, particularly for distant relapse [56, 57] and mortality [53, 54] in the adjuvant setting. Here, presumptive micro-metastases are established prior to definitive surgery in non-breast tissues. MD change could either be a specific surrogate for the influence of ET on non-breast tissues or a broader surrogate for favourable pharmacokinetics such as increased drug activation, longer retention or reduced deactivation. On the former point, there is little cancer-specific data although estrogen and anti-estrogens are well known to influence bone biochemistry, a common metastatic site. On the latter point, a clinically relevant possibility is that MD change may predict tamoxifen activation status. Cytochrome P450 2D6 enzyme (CYP2D6) metabolizer status influences such activation, with poor metabolisers (PMs) showing a degree of tamoxifen resistance. Extensive metabolizers, rather than PMs, were more likely to have a greater than 10% MD reduction in 12 month post treatment mammogram [90] and have been found in some studies to have improved outcomes [91], although results have been inconsistent, possibly due to molecular heterogeneity in other tamoxifen-metabolising enzymes [92]. As a potential marker of tamoxifen activation, MD change bypasses the need both for a detailed understanding of and complex testing for such enzyme variants.

### Insights from the pathobiological links between MD, BC risk and ET action

Reassuringly, a number of known mechanisms of pathogenic importance to BC have been found to be interlinked with MD, including key signalling pathways, enhancers of motility, angiogenesis and inflammation, which further validate MD as intrinsic to BC development. The identification of specific molecules within these functional domains may indicate that these molecules are particularly important to oncogenesis and thereby, could elevate them to suitable drug targets. For example, the TAB2 protein is implicated in disrupting co-repressor binding to ERs in response to tamoxifen, preventing tamoxifen-driven transcriptional suppression [64]. High MD and BC risk linked to polymorphisms in this molecule may arise from an impact on the co-repressor:co-activator balance in normal health, increasing the impact of estrogens on normal tissues including the breast [63]. Attenuating this function could be explored in prevention and treatment in women with high MD.

### MD evaluation modalities and radiation exposure

Various imaging modalities have been developed to evaluate MD, among which X ray-based and MR-based modalities are the best established [93]. On the balance of cost effectiveness and measurement efficiency, digital mammography (DM) is the most commonly utilized in clinical practice, with continuous advancement in MD measurement software. Although the involved radiation risk is generally small, the issue becomes a concern when taking into account that the mammographic examination is taking place in basically asymptomatic woman in the preventative setting. A recent study by Al Kattar et al [94] demonstrated that the mean glandular radiation dose (MGD) delivered from DM as measured in a standard breast phantom was at most 2.05 mGy at an average thickness of 50mm, which is far less than maximal MGD accepted in the AFSSAPS 2006 report and FDA allowable limit of 3 mGy per exposure [95]. From a clinical perspective, the lifetime risk of BC incidence attributed to 2D bilateral screening mammography in 40 year woman has been estimated to be 5-7/100000 [96].

### Is MD ready for prime time?

The promise of MD utility and the lack of timely alternatives have encouraged many researchers to consider MD as a surrogate for risk of BC events [97] and to set it as a secondary [98, 99] or even as a primary endpoint [100] in clinical trials including those evaluating AIs in the preventive setting [101], but is this confidence in the potential of MD change as a prediction tool justified?

The potential for MD to guide BC intervention has long been recognised [102]. As opposed to tissue and circulating biomarkers [103], MD is particularly appealing since it; 1) significantly correlates with both BC risk and outcomes, 2) significantly changes in response to some endocrine manipulations, 3) is non-invasive, and 4) may be easily incorporated in the routine care already employed in screening and follow up of BC; thereby, minimising additional cost. However, a cancer biomarker should not be introduced into clinical utility before it has been validated and before methods and protocols have been optimised for effective widespread usage.

For predictive markers that may change treatment decisions, validation has to be rigorous as described by Prentice *et al* [104] and subsequently emphasized by the Boyd group [105, 106]. The National Cancer Institute of USA has outlined a strategy for biomarker discovery and development consisting of three phases. Phase I incorporates biomarker discovery, assay development and initial small pilot studies, all relatively well covered for MD in BC. Phase II undertakes the study of large independent retrospective cohorts, as reviewed here (Table 3). Phase III involves both the retrospective analysis of material or data from prospective clinical trials as reported here for the IBIS I primary prevention study [48] moving forward to new prospective clinical trials where the biomarker will be employed for decision making in the experimental arm and outcomes are compared with standard practice in the control arm.

Considering the adjuvant scenario where the intuitive utility of MD monitoring is to guide switching between SERM and AI, a retrospective analysis of the BIG 1 study where large cohorts received treatment with AI alone, SERM alone, AI followed by SERM or vice versa [107], appears to be well indicated. If patients with no significant MD change on initial therapy show better outcomes after switching than those with similar MD dynamics randomised to remain on the same initial therapy, this could pave the way for a prospective trial using MD change to tailor therapy type with the potential to improve BC outcomes.

Additionally, before MD change can be introduced as a predictive biomarker into the clinical practice, some more pragmatic knowledge gaps also need to be filled:

- What MDR threshold best predicts outcome improvement?
- What is the most accurate predicting MD parameter i.e. percent density (in terms of area *vs*. volume) *vs*. absolute measures (e.g. dense area) *vs*. categorical density (in terms of BIRADS *vs*. Boyd's *vs*. Wolf's categories)?
- What is the best (accurate and feasible) MD interpretation method i.e. visual *vs*. computer assisted *vs*. fully automated methods?

## MATERIALS AND METHODS

Relevant literature was identified by an interrogation of Embase and Medline electronic databases using the Ovid interface. Our strategy consisted of searching in (all fields), using keywords extracted from the relevant MeSH headings, which were then combined using the Boolean operators, leading to the search terms: [(mammographic density) AND (receptor modulat* OR arzoxifen* OR lasofoxifen* OR raloxifen* OR tamoxifen*)] and [(mammographic density) AND (aromatase inhibit* OR anastrozol* OR letrozol* OR exemestan*)]. Limitations applied were: 1) English literature, 2) publication year (2000 - current). Full text manuscripts of relevant records were assessed for eligibility. Eligible articles were those investigating BC risk or outcomes and MD changes in relation to ETs in the preventive or adjuvant BC scenarios. Additional articles were retrieved either from relevant article bibliographies or from Pubmed for the monitoring biomarker section. Relevant data from an interim analysis of a study by members of our group were also included. A total of 107 eligible publications were identified and used to construct this review.

## CONCLUSIONS

MD change over the ET course, rather than a single MD measurement, can predict risk or outcome modifications in BC preventive and adjuvant settings with certain restrictions. MD reduction is relatively frequent for patients on tamoxifen, with reasonable evidence in both primary and secondary preventative settings that this correlates with risk reduction. The situation with other SERMs and AIs is unclear with further, larger studies required. Analyses in this area should take account of the potential effects of patients entering menopause or stopping HRT, which may confound results. Development of MD as a biomarker appears relatively well advanced through the later Phase II or early Phase III stages of the NCI biomarker development scheme. Retrospective analysis of prospective trials and subsequent employment of MD in future prospective randomised trials is now required to advance the field towards clinical utility. Additionally, studies are also needed to fill the aforementioned knowledge gaps to define the optimum elements of MD to assess and the method of measurement to employ, to ensure reproducibility across health systems. Further research, exploring the pathobiological relation between MD and BC may also assist in new target discovery and the consequent development of novel therapeutics.

## Abbreviations

AI: Aromatase inhibitor
BC: Breast cancer
EGF: Epidermal growth factor
ER: Estrogen receptor
ET: Endocrine therapy
HMD: High mammographic density
LMD: Low mammographic density
IGF 1: Insulin-like growth factor 1
IGF1-R: IGF1 receptor
MD: Mammographic density
MDR: MD reduction
PR: Progesterone receptor
SERM: Selective ER modulator
SNP: Single nucleotide polymorphism.
